# Right and Left-Handedness

**Published:** 1886-02

**Authors:** 


ARTICLE IV.

### RIGHT AND LEFT-HANDEDNESS.

Dr. Chudleigh, in the *British Medical Journal*, writes as follows:

The functional asymmetry which makes one hand stronger than the other must have some anatomical basis which ought to be discoverable; but no one yet seems to have detected the cause. Many a time have I gone over the unilateral structures of the body, to consider if any one of them was calculated to influence its own side for good or evil; but I was always accompanied in my search by this baffling forethought, that if I did succeed in finding some cause for a general excess of muscular nutrition in the right limbs, there would be a corresponding excess of nervous nutrition in the right side of the brain; an advantage which, by the decussation of fibres, would be transferred to the left side of the body, and things would be equal again. Yet, in spite of this difficulty, I have been continually haunted by the idea that it is in the innominate artery, if anywhere, that we are to find the solution of the mystery. The innominate secures for its own subclavian and carotid these advantages over the subclavian and carotid which arise direct from the aorta on the other side. A tube of a given calibre is more effective than two tubes of half that calibre; therefore the innominate, besides serving as a kind of funnel-mouth to catch the stream, is a more effective channel than the subclavian and carotid together would be if arising from the aorta as two distinct tubes.

Again, the innominate lies rather more in a direct line with the aorta than do the two equivalent vessels on the other side; wherefore the innominate would have the advantage of lying more directly in the set of the stream.

And the advantage of being a little nearer the heart, even if infinitesimal, is on the same side. Thus the "bend sinister" of the aorta, and the existence of the innominate artery, tend to increase the blood-supply of the arm and brain on the right side. This fairly accounts for a slight excess of muscular nutrition in the right arm; and a slight excess, by provoking use, would induce further development. But the right carotid has the same advantage as the subclavian; how is it, then, that equilibrium is not restored by an excess of nerve-force being sent across to the left of the body from the right of the brain? When we observe that ponderous muscular power can co-exist with a comparatively small brain, as in the elephant and boa, whereas a large brain with small muscle by no means implies muscular power, it seems fair to conclude that an increase of muscle produces more physical effect than an equal increase of nerve-matter, and that consequently the advantages of the innominate tell more on the muscle of the right arm than on the nerves of the left side of the body. But this only accounts for the superiority of the right arm; what can be said about the remainder of the right side? In the first place, not all the right side is superior to the left; next to the arm, it is mainly in the leg that "dexterity" is discernible. In the next place, the superiority of the right leg is not nearly so marked as that of the right arm, and may be due to the fact that a sense of power in the right arm inspires a sense of confidence in all else that is right, whence results more frequent use, and consequent development. Also the advance of the right arm often necessitates the advance of the right foot, as in sword-exercise. But in numerous other cases the left leg, if left to itself, displays a degree of forwardness which excites a suspicion that its inequality is not natural, but induced. I have asked a considerable number of the London Shoe-brigade which foot their customers usually present first, and the replies have been that the left is first presented almost invariably.

The foregoing theory can be easily tested in several ways. Abnormal cases are occasionally noted where there is no innominate artery, the right subclavian and carotid arising directly from the aorta, like their fellows on the other side. I think that one or more such irregularities were observed last year in the dissecting-room of University College, Liverpool. It would be interesting to learn if there were a history of left-handedness in any of those subjects. Also it sometimes happens that the normal position of viscera is reversed, and all the organs are found to have changed sides. One such case is preserved in the College of Surgeons, and within the last few months another was reported from America. Is it possible to ascertain if left-handedness was observed in either of these?

Some of the facts connected with right and left are curious and interesting. If one offer the right hand to an European adult, the propensity to extend the right hand in return is so strong as to be almost a reflex action. A Bokhara sheik, who suspected Wolff of being a Frank, applied the test of offering him his hand; fortunately for himself, Wolff did not grasp the hand, but responded with a salaam in correct Oriental fashion. But in children, this propensity is either not yet developed, or else is overcome by an innate law of least action. I have tried the experiment of offering my right hand to scores of little children; they invariably give the left, which is nearest, and do not cross the right hand over. If I offer my left, they return the right, again swayed by a law of least action. In sliding on ice, my schoolfellows used to put the right foot forward almost invariably. Though the word Benjamin means Son of a Right Hand, yet the tribe seems to have been notoriously left-handed. The name may have been euphemistic, like Euonumos and Aristera. Von Miklucho Maclay says that Papuans are always left-handed. I should very much like to know something about the Papuan innominate.

